# Distinct biological activities of isomers from several families of branched fatty acid esters of hydroxy fatty acids (FAHFAs)

**DOI:** 10.1016/j.jlr.2021.100108

**Published:** 2021-08-18

**Authors:** Pratik Aryal, Ismail Syed, Jennifer Lee, Rucha Patel, Andrew T. Nelson, Dionicio Siegel, Alan Saghatelian, Barbara B. Kahn

**Affiliations:** 1 Division of Endocrinology, Diabetes and Metabolism, Department of Medicine, Beth Israel Deaconess Medical Center and Harvard Medical School, Boston, MA, USA; 2 Skaggs School of Pharmacy and Pharmaceutical Sciences, University of California San Diego, La Jolla, California, USA; 3 Clayton Foundation Laboratories for Peptide Biology, Salk Institute for Biological Studies, La Jolla, California, USA

**Keywords:** fatty acids, lipids, metabolism, diabetes, adipocytes/obesity, insulin, inflammation, macrophages, stereoisomers, GSIS, 9-OAHPA, 9-oleic acid hydroxy palmitic acid, 9-PAHPA, 9-palmitic acid hydroxy palmitic acid, BMDC, bone marrow–derived dendritic cell, BMDM, bone marrow–derived macrophage, CEL, carboxyl ester lipase, FAHFA, fatty acid esters of hydroxy fatty acid, GPCR, G protein–coupled receptor, GPR40, G protein–coupled receptor 40, GSIS, glucose-stimulated insulin secretion, HSA, hydroxy stearic acid, LPS, lipopolysaccharide, OAHSA, oleic acid hydroxy stearic acid, PAHSA, palmitic acid hydroxy stearic acid, POHSA, palmitoleic acid hydroxy stearic acid, SAHSA, stearic acid hydroxy stearic acid, WAT, white adipose tissue

## Abstract

Branched fatty acid esters of hydroxy fatty acids (FAHFAs) are endogenous lipids with antidiabetic and anti-inflammatory effects. Each FAHFA family consists of esters with different acyl chains and multiple isomers with branch points at different carbons. Some FAHFAs, including palmitic acid hydroxy stearic acids (PAHSAs), improve insulin sensitivity and glucose tolerance in mice by enhancing glucose-stimulated insulin secretion (GSIS), insulin-stimulated glucose transport, and insulin action to suppress hepatic glucose production and reducing adipose tissue inflammation. However, little is known about the biological effects of other FAHFAs. Here, we investigated whether PAHSAs, oleic acid hydroxy stearic acid, palmitoleic acid hydroxy stearic acid, and stearic acid hydroxy stearic acid potentiate GSIS in β-cells and human islets, insulin-stimulated glucose uptake in adipocytes, and anti-inflammatory effects in immune cells. We also investigated whether they activate G protein–coupled receptor 40, which mediates the effects of PAHSAs on insulin secretion and sensitivity in vivo. We show that many FAHFAs potentiate GSIS, activate G protein–coupled receptor 40, and attenuate LPS-induced chemokine and cytokine expression and secretion and phagocytosis in immune cells. However, fewer FAHFAs augment insulin-stimulated glucose uptake in adipocytes. S-9-PAHSA, but not R-9-PAHSA, potentiated GSIS and glucose uptake, while both stereoisomers had anti-inflammatory effects. FAHFAs containing unsaturated acyl chains with higher branching from the carboxylate head group are more likely to potentiate GSIS, whereas FAHFAs with lower branching are more likely to be anti-inflammatory. This study provides insight into the specificity of the biological actions of different FAHFAs and could lead to the development of FAHFAs to treat metabolic and immune-mediated diseases.

Type 2 diabetes (T2D) is a global epidemic with 463 million adults affected (1). The higher incidence of T2D in childhood is likely to result in earlier onset of complications such as cardiovascular and renal diseases (2) and could shorten the life span for the first time in more than a century (3). T2D is characterized by impaired function of the islet β-cells that secrete insulin, and resistance to the actions of insulin in peripheral tissues (i.e., insulin resistance). Despite recent advances in understanding the molecular mechanisms contributing to T2D and the development of new treatment modalities, the medical management of T2D remains inadequate (4).

There is increasing evidence that bioactive lipids play key roles in disease processes (5, 6). We discovered a novel class of endogenous mammalian lipids, branched fatty acid esters of hydroxy fatty acids (FAHFAs). One family of FAHFAs, palmitic acid hydroxy stearic acids (PAHSAs), has antidiabetic and anti-inflammatory effects (7). Yore *et al.* (7) initially identified more than 16 FAHFA families characterized by different fatty acid and hydroxy fatty acid combinations. Each FAHFA family has at least 6–8 different isomers defined by the position of the ester bond between the acyl chains (7). Recently, Zhu *et al.* (8) identified 51 different FAHFA families, including 301 regioisomers in mouse visceral adipose tissue. The number of FAHFA families and regioisomers in mice increased with age (8). Seventeen different FAHFA families and 64 regioisomers were identified in the white adipose tissue (WAT) of the golden hamster (9). In human WAT, there are 583 FAHFA regioisomers from 21 FAHFA families (10). In addition, FAHFAs are present in foods commonly consumed by humans (7, 11). In rice and *Arabidopsis thaliana*, Zhu *et al.* (12) identified 49 potential FAHFA families, including 262 regioisomers. Analysis of different foods with antioxidative, anti-inflammatory, and beneficial metabolic effects revealed that stearic acid hydroxy stearic acid (SAHSA) was the most abundant FAHFA in these foods (11). Thus, there are hundreds of structurally distinct FAHFAs in mammals and plants, most of which have not been characterized in terms of biological activities.

Multiple isomers of one FAHFA family, PAHSAs, are lower in serum and subcutaneous adipose tissue of insulin-resistant humans compared to insulin-sensitive humans (7). PAHSA levels correlate strongly with insulin sensitivity as measured by euglycemic clamps in humans (7). Administration of 5- or 9-PAHSA to aged chow-fed mice and high-fat diet (HFD)-fed mice improves glucose tolerance, and systemic and hepatic insulin sensitivity without altering the body weight (7, 13, 14). The improvement in HFD-fed mice is due, in part, to PAHSA enhancement of insulin action to suppress lipolysis in adipose tissue (14). 5- and 9-PAHSAs also augment glucose-stimulated insulin and glucagon-like peptide-1 secretion in young chow-fed mice and mice with insulin resistance due to aging but not in HFD-fed mice (7, 13). 9-PAHSA also improves diabetes-related cognitive impairment in mice (15). In addition to PAHSAs, long-term, high intake of other FAHFA family members, 9-palmitic acid hydroxy palmitic acid (9-PAHPA) and 9-oleic acid hydroxy palmitic acid (9-OAHPA), augments basal metabolism and insulin sensitivity in “healthy” mice (16) and in mice on an HFD and high-sugar diet (17). Although some healthy mice developed hepatic steatosis when supplemented with 9-PAHPA or 9-OAHPA (16), no adverse hepatic effects were observed in high fat– and high sugar–fed mice treated with 9-PAHPA or 9-OAHPA (16, 17).

PAHSAs have direct effects in a number of cell and tissue types. In human pancreatic islets from both nondiabetic people and people with T2D, PAHSAs augment glucose-stimulated insulin secretion (GSIS) (7, 13, 18). They also directly enhance glucagon-like peptide-1 secretion from gut enteroendocrine cells (7). In human preadipocytes, both 5- and 9-PAHSA augment adipocyte differentiation (19). In 3T3-L1 adipocytes, PAHSAs potentiate insulin-stimulated glucose uptake and GLUT4 glucose transporter translocation to the plasma membrane (7).

The anti-inflammatory effects of PAHSAs include attenuation of proinflammatory cytokine production by adipose tissue macrophages in insulin-resistant obese mice and reduction of lipopolysaccharide (LPS)-induced dendritic cell activation and subsequent proinflammatory cytokine production in vitro (7, 20). In addition, PAHSAs have anti-inflammatory effects in the gut and protect mice from experimental colitis by decreasing colonic inflammation (21). In nonobese diabetic mice, an autoimmune model of type 1 diabetes, chronic administration of 5- and 9-PAHSAs delays the onset and reduces the incidence of type 1 diabetes by attenuating immune responses and exerting direct protective effects on β-cell survival and function (22). The omega-3-fatty acid-containing FAHFA, 13-docosahexaenoic acid hydroxy linoleic acid, and the omega-6-fatty acid–containing FAHFA, 13-linoleic acid hydroxy linoleic acid, also prevent LPS-induced expression and secretion of proinflammatory cytokines from macrophages (20, 23). Whether isomers of other FAHFA families are anti-inflammatory is not known.

There is limited information about whether the position of the ester bond or the degree of unsaturation of the acyl chains in different FAHFAs affects biological activity although these factors affect susceptibility to hydrolysis (24). For example, carboxyl ester lipase (CEL), an FAHFA hydrolase, preferentially hydrolyzes FAHFAs with the ester bond further away from the carboxylate (12-PAHSA > 9-PAHSA ≫ 5-PAHSA) (24). We also found that FAHFAs containing unsaturated acyl chains were hydrolyzed more quickly than FAHFAs containing saturated fatty acids (i.e., palmitoleic acid hydroxy stearic acids [POHSAs]/oleic acid hydroxy stearic acid [OAHSAs] > PAHSAs/SAHSAs) (24). However, potential effects of these structural characteristics on FAHFA biological activities are not known. Therefore, the first aim of this study was to determine whether other PAHSA isomers and isomers of other FAHFA families—POHSA, OAHSA, and SAHSA—have biological effects to potentiate GSIS in islet cells, glucose uptake in adipocytes, and anti-inflammatory effects in dendritic cells and macrophages.

The second aim is to determine whether FAHFA stereoisomers have preferential biologic effects. Based on the configuration of the stereogenic branching carbon, FAHFAs can possess either R or S configuration. Both enantiomers of 9-PAHSA were identified in mouse WAT (25), with the R enantiomer being more abundant. Both enantiomers of 5-PAHSA were found in human and murine breast milk (26), with the majority of 5-PAHSA possessing the R configuration (26). Paluchova *et al.* (27) found that both 13(R)- and 13(S)-DHAHLA enantiomers were present in mouse adipose tissue and 13(S)-DHAHLA is the major isomer. There is little information on the functional differences in these enantiomers. Nelson *et al.* (25) showed that CEL selectively hydrolyzes S-9-PAHSA over R-9-PAHSA. In addition, the biosynthesis of R-9-PAHSA is favored over S-9-PAHSA, suggesting stereospecific production and degradation of FAHFAs. However, this may not be the case for biologic effects. Both stereoisomers of 13-DHAHLA inhibited antigen and prostaglandin E2 (PGE2)-induced chemotaxis and degranulation of mast cells (27). Therefore, we sought to determine whether the beneficial effects of 9-PAHSA on GSIS, glucose uptake, and anti-inflammatory effects are enantiomer specific. We also determined whether different FAHFAs and stereoisomers activate G protein–coupled receptor 40 (GPR40), which we previously showed mediates some of the beneficial effects of PAHSAs (13). This information will be useful to understand the biological roles of FAHFAs and how to develop them into therapeutic agents.

## Materials and methods

### Materials and reagents

The following FAHFAs were obtained from Cayman Chemical (Ann Harbor, MI): 5-PAHSA (Cat# 17043), 9-PAHSA (Cat# 17037), 10-PAHSA (Cat# 19973), 12-PAHSA (Cat# 17107), 13-PAHSA (Cat# 17044), 5-POHSA (Cat# 17114), 9-POHSA (Cat# 17040), 10-POHSA (Cat# 19974), 12-POHSA (Cat# 17105), 13-POHSA (Cat# 17111), 5-OAHSA (Cat# 17115), 9-OAHSA (Cat# 17039), 10-OAHSA (Cat# 19975), 12-OAHSA (Cat# 17108), 13-OAHSA (Cat# 17112), 5-SAHSA (Cat# 17113), 9-SAHSA (Cat# 17109), 10-SAHSA (Cat# 19976), 12-SAHSA (Cat# 17106), and 13-SAHSA (Cat# 17110). R and S enantiomers of 9-PAHSA were synthesized as previously described (25). Concentrations of FAHFAs in all biological assays is 20 μM, concentration of DMSO is 0.01% unless mentioned otherwise. The Human Insulin ELISA kit (Cat#80-INSHU-E01.1) was purchased from ALPCO (Salem, NH). The Mouse Insulin ELISA kit (Cat# 90080) was obtained from Crystal Chem (Elk Grove Village, IL). Mouse Tnfα (Cat# 430901) and Il-6 (Cat# 431301) ELISA kits were obtained from BioLegend (San Diego, CA). [3H]Deoxy-glucose (NET328A) was obtained from PerkinElmer (Waltham, MA). All the other chemicals were purchased from Sigma-Aldrich (St Louis, MO) unless otherwise stated.

### Human islets and clonal pancreatic β-cells

Human islets from nondiabetic donors were obtained from Prodo Laboratories (Irvine, CA). The detailed demographic information of the islet donors is listed in Table 1. Clonal pancreatic MIN6 β-cells were obtained from the ATCC (CRL 11506).

**Table 1:** Demographic information for human islet donors

Human Donor	Donor 1	Donor 2	Donor 3	Donor 4	Donar 5	Donor 6
Source	Prodo Labs	Prodo Labs	Prodo Labs	Prodo Labs	Prodo Labs	Prodo Labs
Age (years)	43	29	48	45	42	48
Race/Ethnicity	Asian	Hispanic	African American	African American	Caucasian	Caucasian
Sex	Female	Female	Male	Male	Male	Male
Height (Inches)	59	65	73	72	75	70
Weight (pounds)	115	157	157	235	224	179
BMI	22.8	26.2	20.2	31.5	27.97	25.6
History of diabetes	No	No	No	No	No	No
HbA1C (%)	5.4	5.1	5.2	5.8	4.9	5.9
Cause of death	Stroke	Stroke	Stroke	Anoxic	Head trauma	Anoxic
Islet purity (%)	95	95	95	95	90	95
Islet viability (%)	95	95	95	95	95	95

All human islets used were obtained from Prodo Labs. Demographic information for six different donors and percent viability and purity of islet preparations are provided.

### GSIS studies

MIN6 cells were maintained at 37°C and cultured in RPMI 1640 medium containing 10% heat-inactivated FBS supplemented with 100 IU/ml penicillin and 100 IU/ml streptomycin, 1 mM sodium pyruvate, 50 mM 2-mercaptoethanol, and 10 mM Hepes (pH 7.4). The medium was changed twice a week, and cells were subcloned weekly. MIN6 cells and human islets were serum-starved using KRB buffer for 3 h and treated with FAHFAs (20 μM) or DMSO (0.01%) for the last 1 h. GSIS studies were performed as described (28).

### Measurement of GPR40-associated Ca^2+^ currents

Calcium measurements were performed by Multispan Inc. (Hayward, CA). Mouse GPR40 stable cell line and HEK293T cells were grown in DMEM, 10% FBS, and 1 mg/ml puromycin. On the day of assay, cells were lifted with nonenzymatic cell stripper and seeded in 384-well poly-d-lysine–coated plate in the assay buffer (HBSS + 20 mM Hepes) at an appropriate density. The calcium dye loading buffer (Multispan, Cat# MSCA01-1) was added to cells and incubated for 30 min at 37°C followed by 30 min at room temperature. In the antagonist mode, cells were incubated with racemic 9-PAHSA, R-9 PAHSA, or S-9 PAHSA at different concentrations. Calcium flux was monitored for 180 s with FAHFAs injected into the well at the 19th second read by FLIPR 384 (Molecular Devices, San Jose, CA).

### Generation and treatment of bone marrow–derived dendritic cells and bone marrow–derived macrophages

Bone marrow–derived dendritic cells (BMDCs) and bone marrow–derived macrophages (BMDMs) were generated as previously described (29). Cells were incubated with FAHFAs (20 μM) or DMSO (0.01%) 10 min before LPS (100 ng/ml) stimulation. Twenty four hours after LPS stimulation, cell culture media was collected for cytokine measurements. Tnfα and Il-6 levels were measured using mouse ELISA kits according to manufacturer's protocol (BioLegend, San Diego, CA).

### Quantitative PCR

RNA was extracted using TRI Reagent (MRC, OH), and cDNA was generated with random hexamers (Clontech, CA). Quantitative real-time PCR was performed with the ABI Prism sequence detection system. Each sample was run in duplicate, and the quantity of mRNA in each sample was normalized to mouse TATA-box binding protein mRNA levels. All primers were obtained from IDT, Iowa. Primer sequences are as follows: *Tnfα* forward 5′- GATCGGTCCCCAAAGGGATG, *Tnfα* reverse 5′- GGTGGTTTGTGAGTGTGAGG, *Il-6* forward 5′- TCCTCTCTGCAAGAGACTTCC, *Il-6* reverse 5′- TTGTGAAGTAGGGAAGGCCG, *Il-1β* forward 5′- GAA ATG CCA CCT TTT GAC AGT G-3′, *Il-1β* reverse 5′ TGG ATG CTC TCA TCA GGA CAG -3′, *Ccl2* forward 5′-TAA AAA CCT GGA TCG GAA CCA AA-3′, *Ccl2* reverse 5′- GCA TTA GCT TCA GAT TTA CGGG GT-3′, *Ccl3* forward 5′- TGT ACC ATG ACA CTC TGC AAC-3′, *Ccl3* reverse 5′- CAA CGA TGA ATT GGC GTG GAA -3′, *Ccl5* forward 5′- TTT GCC TAC CTC TCC CTC G-3′, and *Ccl5* reverse 5′ – CGA CTG CAA GAT TGG AGC ACT -3′.

### Phagocytosis assay

Phagocytosis was performed according to the manufacturer's protocol (Abcam, Cambridge, MA, cat # ab211156). BMDMs were treated with DMSO, FAHFAs (20 μM), or control fatty acids (20 μM) for 15 min before LPS (100 ng/ml) for 24 h. Prelabeled zymosan particles were used as the pathogen for triggering phagocytosis. The engulfed zymosan particles react with the substrate to produce a colorimetric signal that can be detected by absorbance at 405 nm.

### Glucose uptake studies in differentiated 3T3L1 adipocytes

3T3-L1 fibroblasts were cultured and differentiated as previously described (30). Insulin-stimulated glucose uptake studies in differentiated 3T3-L1 adipocytes were initiated on day 8 of differentiation after 24-h pretreatment with FAHFAs at 20 μM or vehicle (DMSO, 0.01%). 3T3 L1 adipocytes were then serum-starved for 3 h and after 20 min of incubation with the Krebs-Ringer-Hepes buffer (7), adipocytes were stimulated with 10 nM insulin for 20 min. After that, cells were incubated with 1 μCi of [3H]deoxy-glucose for 10 min. At the end of the experiment, cells were washed 3 times with the Krebs-Ringer-Hepes buffer supplemented with 200 mM glucose and 10 μM cytochalasin B to remove unincorporated radiolabeled [3H]deoxy-glucose. Cells were then lysed with 0.5% SDS, and radioactivity of the lysed cells was measured in CytoScint scintillation liquid in the scintillation counter. Protein concentration was measured by the Bicinchoninic Acid Protein Assay (Thermo Scientific, Waltham, MA).

### Mouse studies

All animal procedures were conducted in compliance with protocols approved by IACUC. Male C57 BL6 mice were obtained from Jackson laboratory and were singly housed in ventilated cages. Mice were placed on an HFD (Research Diets, TD. 93075) at 5 weeks of age. After 11 weeks on an HFD, mice were implanted with subcutaneous mini pumps (ALZET, Model 2006) delivering either vehicle (50% PEG-400%, 0.5% Tween-80%, 49.5% distilled water) or 12 OAHSA (200 μl volume with a flow rate of 0.15 μl/h) for 151 days. Pumps were changed every 42 days. Body weight and food intake were measured weekly, and the body composition was measured by MRI. Insulin tolerance test (0.65 U/kg body weight) and oral glucose tolerance test (1.5 g/kg body weight) were performed as described (7) after 5 h food removal. Serum was collected in the ad lib–fed state in mice that had been treated with 12 OAHSA for 151 days.

### OAHSA synthesis and measurement

12-OAHSA was synthesized as described (31). Serum 9-OAHSA and 12-OAHSA levels were measured as described (32) with an internal standard and transitions targeted for OAHSA m/z 563.5 → 281.2 and m/z 563 → 299.2.

## Results

### Several PAHSA, POHSA, OAHSA, and SAHSA isomers potentiate GSIS

**Fig. 1:**
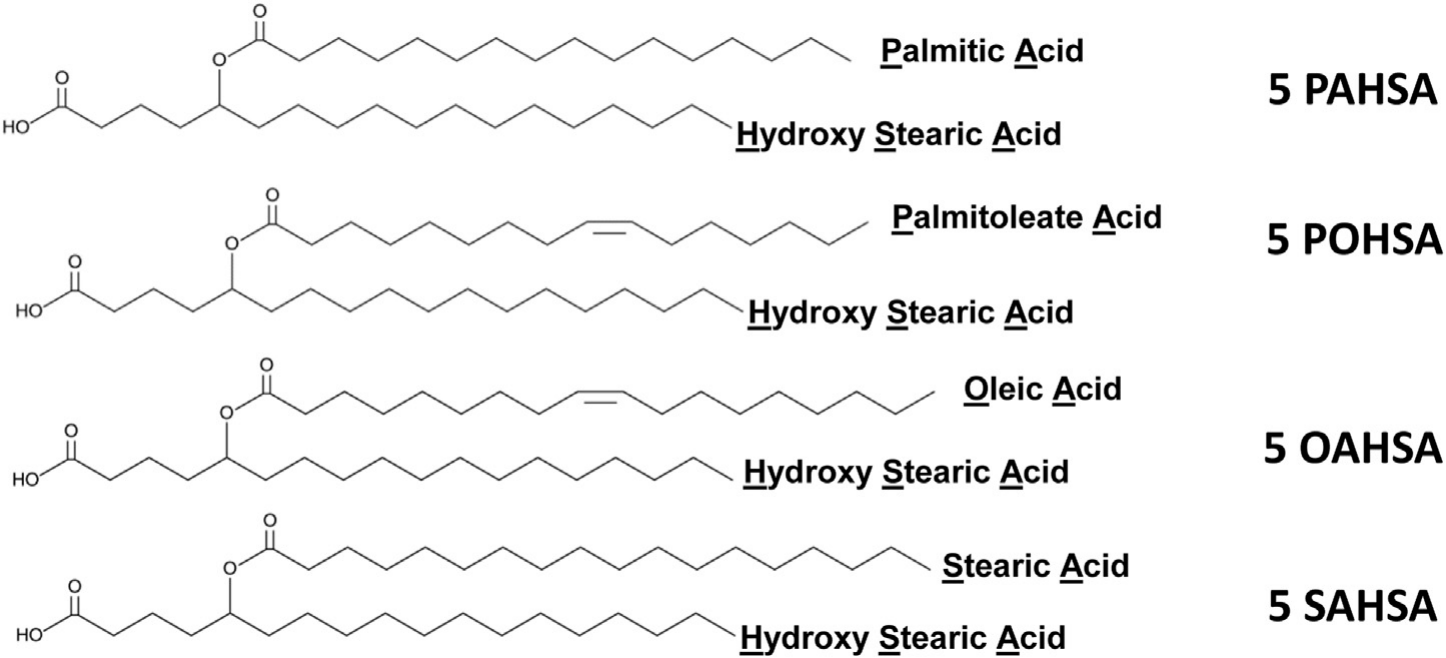
Structure of selected 5 FAHFA isomers. Palmitic acid hydroxystearic acid (PAHSA), palmitoleic acid hydroxystearic acid (POHSA), oleic acid hydroxystearic acid (OAHSA), and stearic acid hydroxystearic acid (SAHSA). FAHFA, fatty acid ester of hydroxy fatty acid.

**Fig. 2:**
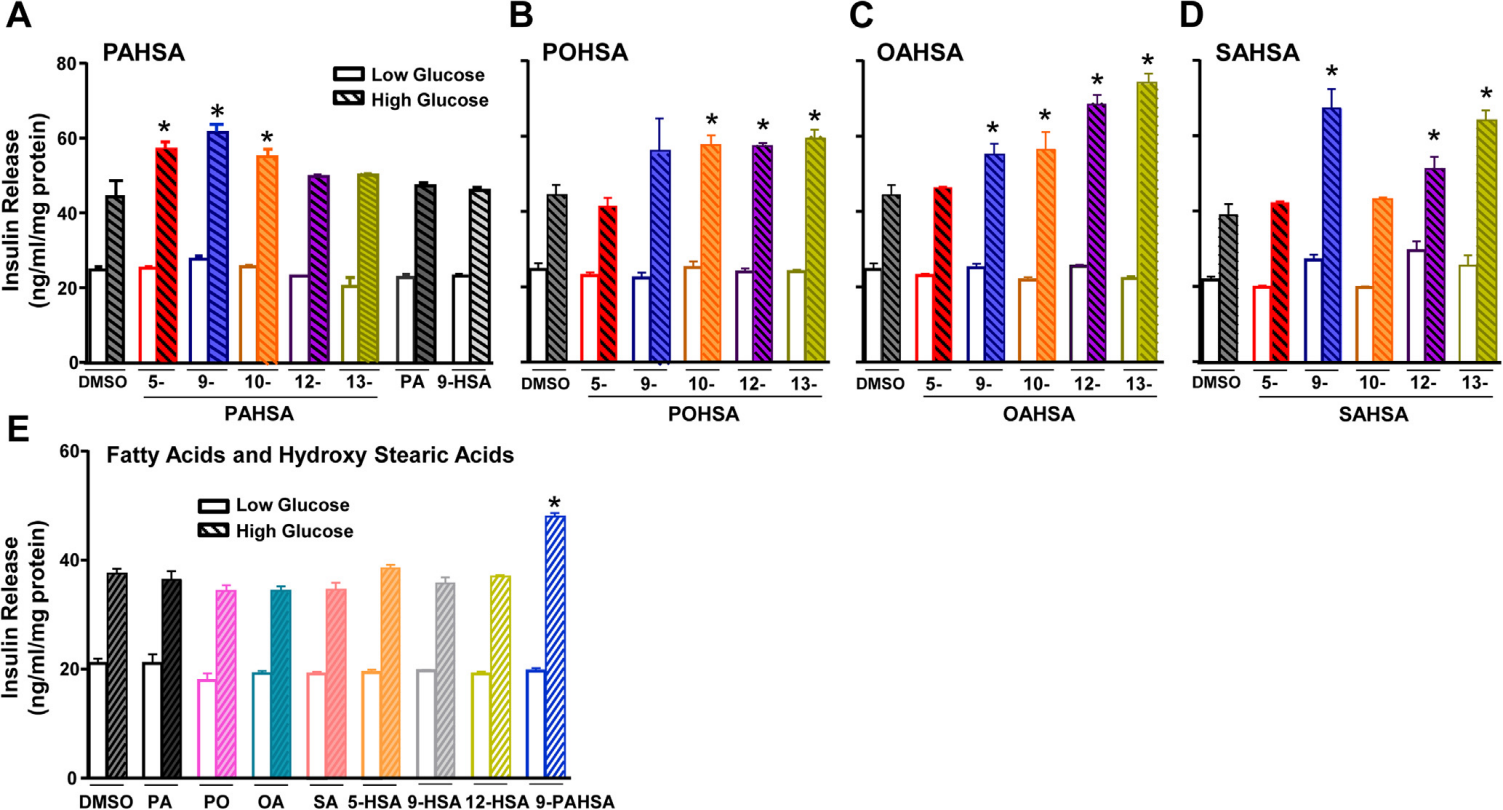
Glucose-stimulated insulin release from MIN6 cells. Many FAHFA isomers potentiate glucose-stimulated insulin secretion in clonal pancreatic β-cells, MIN6 cells. MIN6 cells were incubated with DMSO (0.1%, control) or FAHFAs (20 μM) for 1 h and then stimulated with low (2.5 mM) or high (20 mM) concentrations of glucose for 45 min. n = 6–8 wells/condition. Data are the means ± SEM. Each isomer was tested in 2–3 independent studies. ∗*P* < 0.05 versus DMSO with 20 mM glucose; one-way ANOVA. A: 5-, 9-, 10-, 12-, and 13-PAHSA, palmitic acid (PA), and 9-hydroxy stearic acid (HSA); (B) 5-, 9-, 10-, 12-, and 13-POHSA; (C) 5-, 9-, 10-, 12-, and 13-OAHSA; (D) 5-, 9-, 10-, 12-, and 13-SAHSA; (E) palmitic acid, palmitoleic acid (PO), oleic acid (OA), stearic acid (SA), and hydroxy stearic acids (5-, 9-, and 12-HSA). FAHFA, fatty acid ester of hydroxy fatty acid; PAHSA, palmitic acid hydroxy stearic acid; POHSA, palmitoleic acid hydroxy stearic acid; OAHSA, oleic acid hydroxy stearic acid; SAHSA, stearic acid hydroxy stearic acid.

**Fig. 3:**
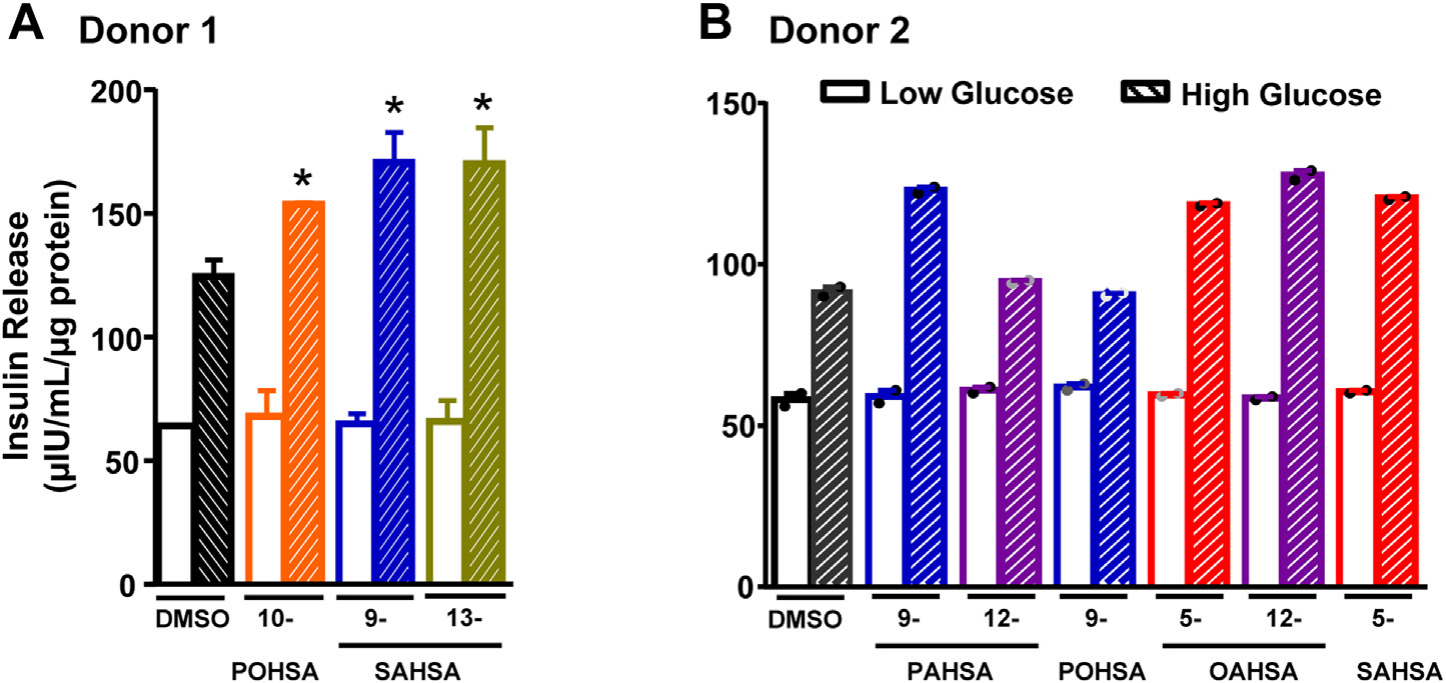
Glucose-stimulated insulin release from human islets. Many FAHFA isomers potentiate glucose-stimulated insulin secretion in isolated human islets. Insulin secretion was performed in primary human islets from two independent donors. Islets were incubated with low (2.5 mM) or high (20 mM) concentrations of glucose ex vivo in the presence of FAHFAs (10-POHSA, 9-SAHSA, 13-SAHSA, 9-PAHSA, 12-PAHSA, 9-POHSA, 5-OAHSA, 12-OAHSA and 5-SAHSA) (20 μM) or DMSO. A: GSIS from isolated human islets from donor 1. n = 50 islets/well and three wells per condition, ∗*P* < 0.05 versus DMSO with 20 mM glucose; one-way ANOVA. B: GSIS from isolated human islets from donor 2. n = 50 islets/well and two wells per condition. Statistical analysis was not performed because of n-2 wells per condition. FAHFA, fatty acid ester of hydroxy fatty acid; GSIS, glucose-stimulated insulin secretion; PAHSA, palmitic acid hydroxy stearic acid; POHSA, palmitoleic acid hydroxy stearic acid; OAHSA, oleic acid hydroxy stearic acid; SAHSA, stearic acid hydroxy stearic acid.

Both 5- and 9-PAHSA potentiate GSIS in clonal pancreatic β-cells (MIN6) and in human islets (7, 13). To determine whether other FAHFAs have these effects, we tested all the FAHFAs that were commercially available at the beginning of this project (Fig. 1) to determine whether they potentiate GSIS both in MIN6 cells and human islets (Figs. 2 and 3). The structures of these FAHFAs are shown in Fig. 1.

None of the FAHFAs we tested potentiated GSIS in the presence of a low glucose concentration (2.5 mM, Fig. 2A–D) in MIN6 cells, which is advantageous because augmentation of insulin secretion at a low glucose concentration could cause serious hypoglycemia (7). Within the PAHSA subfamily, the isomers 5-, 9-, and 10-PAHSAs increased insulin secretion by 22%–36% in the presence of a high glucose concentration (20 mM, Fig. 2A). Notably, neither palmitic acid nor 9-hydroxy stearic acid (HSA) alone used at the same concentration as the PAHSAs potentiated GSIS, indicating that the intact PAHSA molecule is required to augment insulin secretion in response to a high glucose concentration. 10-, 12-, and 13-POHSAs increased GSIS by 29%–34%, whereas 5- and 9-POHSA did not augment GSIS at a high glucose concentration (Fig. 2B).

9-, 10-, and 12-OAHSAs also increased GSIS in the presence of high glucose by 25%, 27%, and 54%, respectively, compared with vehicle (DMSO) alone, whereas 5-OAHSA did not affect GSIS. 13-OAHSA increased GSIS by 68% and was the most potent OAHSA in stimulating insulin secretion (Fig. 2C). 9-, 12-, and 13-SAHSAs increased GSIS by 70%, 29%, and 63%, respectively, whereas 5- and 10-SAHSA did not potentiate GSIS (Fig. 2D). For OAHSAs, the extent of potentiation of GSIS may depend on the position of the ester bond connecting the OA and HSA moieties because the OAHSAs with branching distal to the carboxylate head group appear to stimulate insulin secretion the most. This is not apparent for other FAHFA families we tested. Palmitic acid, palmitoleic acid, oleic acid, and stearic acid alone did not potentiate GSIS either at low or high glucose concentration (Fig. 2E). Similarly, none of the HSAs tested, 5-, 9-, and 12 HSA, potentiated GSIS (Fig. 2E). Taken together, these data strengthen the importance of an intact FAHFA molecule to potentiate GSIS in clonal beta cells. They demonstrate that some, but not all FAHFAs, potentiate GSIS and, overall, this does not depend on the saturation of the acyl chain lipids.

We also performed GSIS studies in human islets from two different healthy donors (Fig. 3A, B; donors 1 and 2 in Table 1). We tested only the FAHFA isomers that potentiated GSIS in MIN6 cells because of limited availability of human islets. None of the tested FAHFAs potentiated GSIS at a low glucose concentration (2.5 mM, Fig. 3A, B). At a high glucose concentration (20 mM), 9-PAHSA, 10-POHSA, 5-OAHSA, 12-OAHSA, 5-SAHSA, 9-SAHSA, and 13-SAHSA potentiated GSIS. As a negative control, we included 12-PAHSA and 9-POHSA, which did not potentiate GSIS in MIN6 cells. Similar to MIN6 cells, 12-PAHSA and 9-POHSA did not potentiate GSIS in human islets at a high glucose concentration (20 mM).

### Many FAHFA isomers activate GPR40

**Fig. 4:**
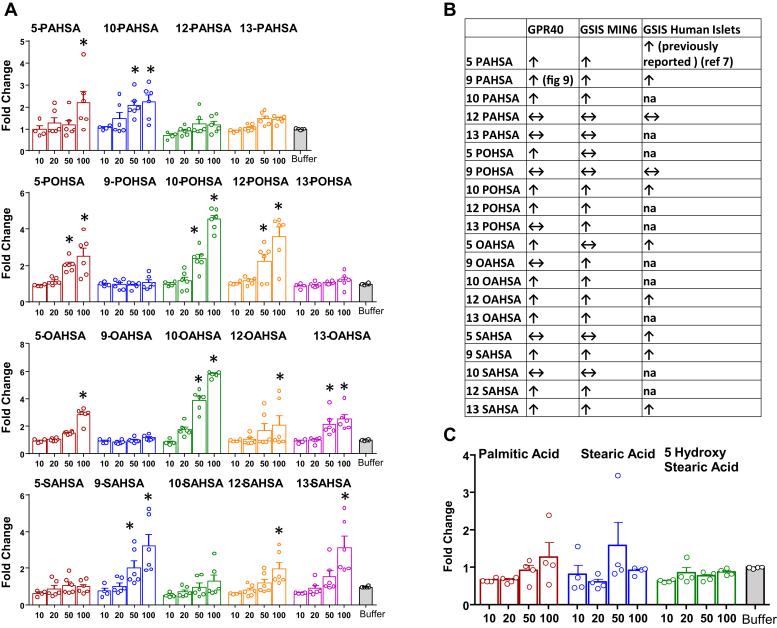
HEK293T mouse GPR40 FLIPR, calcium assay agonist mode with FAHFAs. Many but not all FAHFA isomers activate mouse GPR40 in HEK293T cells. Activation of mouse GPR40 in HEK293T cells by various FAHFA isomers (A). Data are the means ± SEM. n = 4–6; ∗*P* < 0.05 versus buffer; one-way ANOVA. B: Comparison of GPR40 activation (at 50 or 100 μM) in HEK293T cells with GSIS (at 20 μM) in human islets and clonal pancreatic β-cells, MIN6 cells. C: GPR40 is not activated by palmitic acid, stearic acid, or 5-hydroxy stearic acid in HEK293T. Data are the means ± SEM n = 4. FAHFA, fatty acid ester of hydroxy fatty acid; GPR40, G protein–coupled receptor 40; GSIS, glucose-stimulated insulin secretion.

We previously showed that 9-PAHSA dose-dependently activates GPR40 and that inhibition of GPR40 blocks the beneficial effects of 9-PAHSA on GSIS in beta cells and human islets and reduces the effects on glucose tolerance and insulin sensitivity in vivo (13). To determine whether other FAHFA family members activate GPR40, we measured mouse GPR40 activation in stably transfected HEK293T cells. Many FAHFA isomers, but not all, from all four subfamilies we tested activate GPR40 (Fig. 4A). We next compared GPR40 activation by different FAHFAs with GSIS in human islets and MIN6 cells (Fig. 4B). Most of the FAHFAs that activated GPR40 potentiated GSIS in human islets and MIN6 cells with a few exceptions. Notably, individual acyl chain constituents of FAHFAs, that is, palmitic acid, stearic acid, and 5-HSA did not activate GPR40 when used at similar concentrations as the FAHFAs, indicating that the intact FAHFA is required for GPR40 activation (Fig. 4C).

### FAHFA isomers have anti-inflammatory effects

**Fig. 5:**
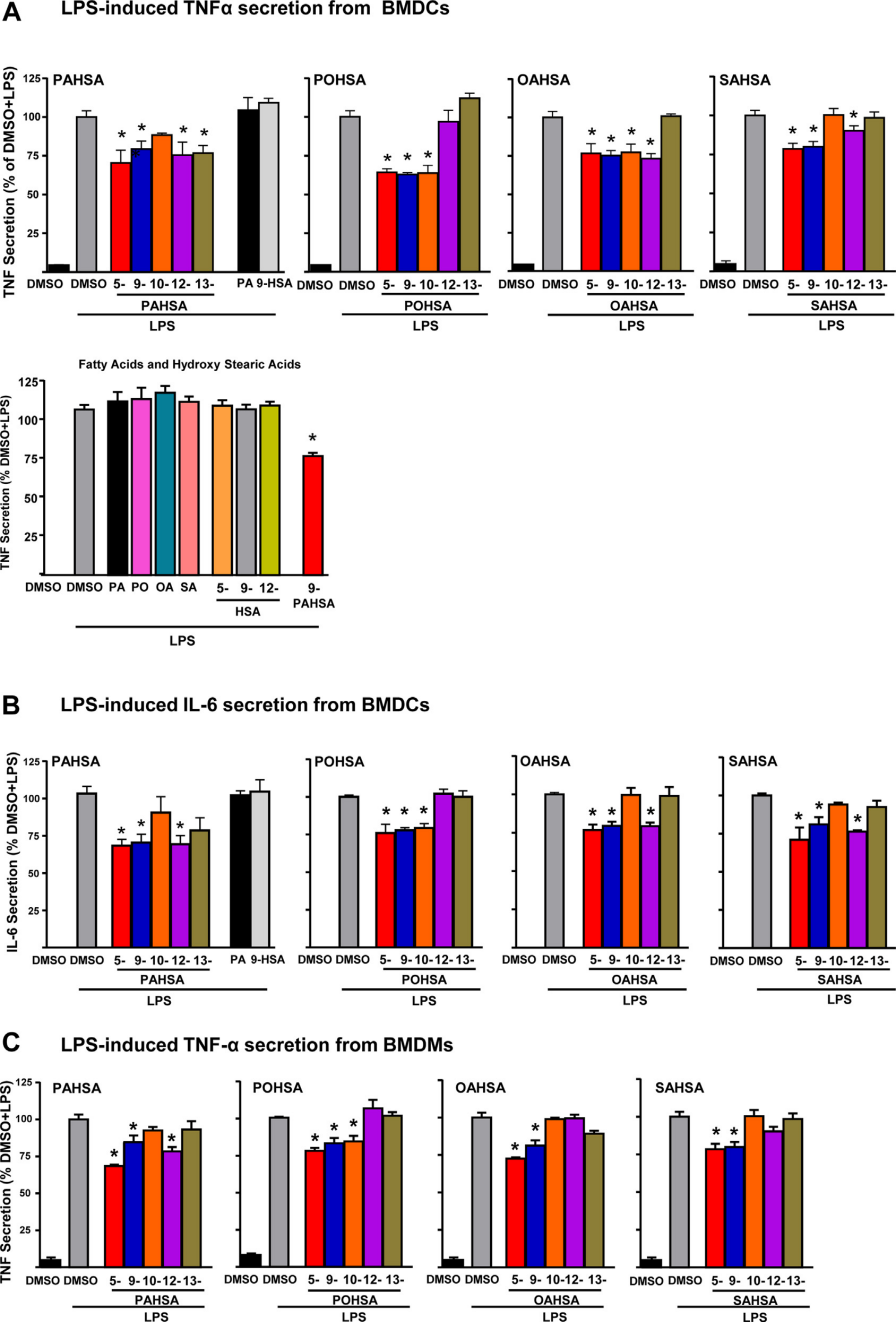
FAHFA isomers inhibit LPS-induced cytokine secretion from BMDMs and BMDCs: (A) FAHFA isomer (20 μM) effects on LPS-induced Tnfα secretion from BMDCs. Last panel shows the lack of effect of individual acyl chain constituents of FAHFA molecules studied as controls. Palmitic acid (20 μM), palmitoleic acid (20 μM), oleic acid (20 μM), stearic acid (20 μM), and hydroxy stearic acids (5-, 9-, and 12-HSA, 20 μM). B: FAHFA effects on LPS-induced Il-6 secretion from BMDCs. C: FAHFA effects on LPS-induced Tnfα secretion from BMDMs. n = 6–8 wells/condition. Each isomer was tested in 2–4 independent studies. Data are the means ± SEM. ∗*P* < 0.05 versus LPS-induced DMSO by one-way ANOVA. BMDC, bone marrow–derived dendritic cell; BMDM, bone marrow–derived macrophage; FAHFA, fatty acid ester of hydroxy fatty acid; LPS, lipopolysaccharide.

Some FAHFAs have been shown to be anti-inflammatory (7, 20, 21, 22, 23, 27), but it is not known whether all FAHFAs have these effects. We investigated whether isomers within other FAHFA families (POHSAs, OAHSAs, and SAHSAs) exert anti-inflammatory effects in BMDCs and BMDMs (Fig. 5)

#### Effects of FAHFAs on LPS-induced Tnf-α secretion

5-PAHSA, 9-PAHSA, 12-PAHSA, 5-POHSA, 9-POHSA, 10-POHSA, 5-OAHSA, 9-OAHSA, 5-SAHSA, and 9-SAHSA attenuated LPS-induced Tnf-α secretion in both BMDCs and BMDMs (Fig. 5A, C), similar to the results with 9-PAHSA (7). However, 13-PAHSA, 10-OAHSA, 12-OAHSA, and 12-SAHSA reduced LPS-induced Tnf-α secretion in BMDCs but not in BMDMs. 10-PAHSA, 12-POHSA, 13-POHSA, 13-OAHSA, 10-SAHSA, and 13-SAHSA do not have any effect on LPS-induced Tnf-α secretion in either BMDCs or BMDMs. None of the individual acyl chain constituents of these lipids, palmitic acid, palmitoleic acid, oleic acid, stearic acid, and HSAs (5-, 9-, and 12-HSA) alone had any effect on LPS-induced Tnf-α secretion in BMDCs (Fig. 5A).

#### Effects of FAHFAs on LPS-induced Il-6 secretion

In BMDMs, 5-PAHSA, 9-PAHSA, 12-PAHSA, 5-POHSA, 9-POHSA, 10-POHSA, 5-OAHSA, 9-OAHSA, 12-OAHSA, 5-SAHSA, 9-SAHSA, and 12-SAHSA attenuate LPS-induced Il-6 secretion in BMDMs (Fig. 5B). 10-PAHSA, 13-PAHSA, 12-POHSA, 13-POHSA, 10-OAHSA, 13-OAHSA, 10-SAHSA, and 13-SAHSA do not have any effect on LPS-induced Il-6 secretion in BMDMs. Taken together, these data demonstrate that many but not all FAHFAs have anti-inflammatory effects. In addition, not all isomers in a specific family have anti-inflammatory effects and this differs among families. These anti-inflammatory effects do not depend on the presence of a particular fatty acyl species. However, LPS-induced Tnf-α secretion in BMDCs and BMDMs and Il-6 secretion in BMDCs is attenuated by all of the 5- and 9-isomers in the FAHFA families tested, suggesting that FAHFAs with lower branching from the carboxylate head group are more reliably anti-inflammatory in these cells than FAHFAs, with higher branching from the carboxylate head group. Some other FAHFAs exert anti-inflammatory effects on one type of antigen-presenting cell and not the other.

### Effects of FAHFAs on LPS-induced chemokine and cytokine gene expression

**Fig. 6:**
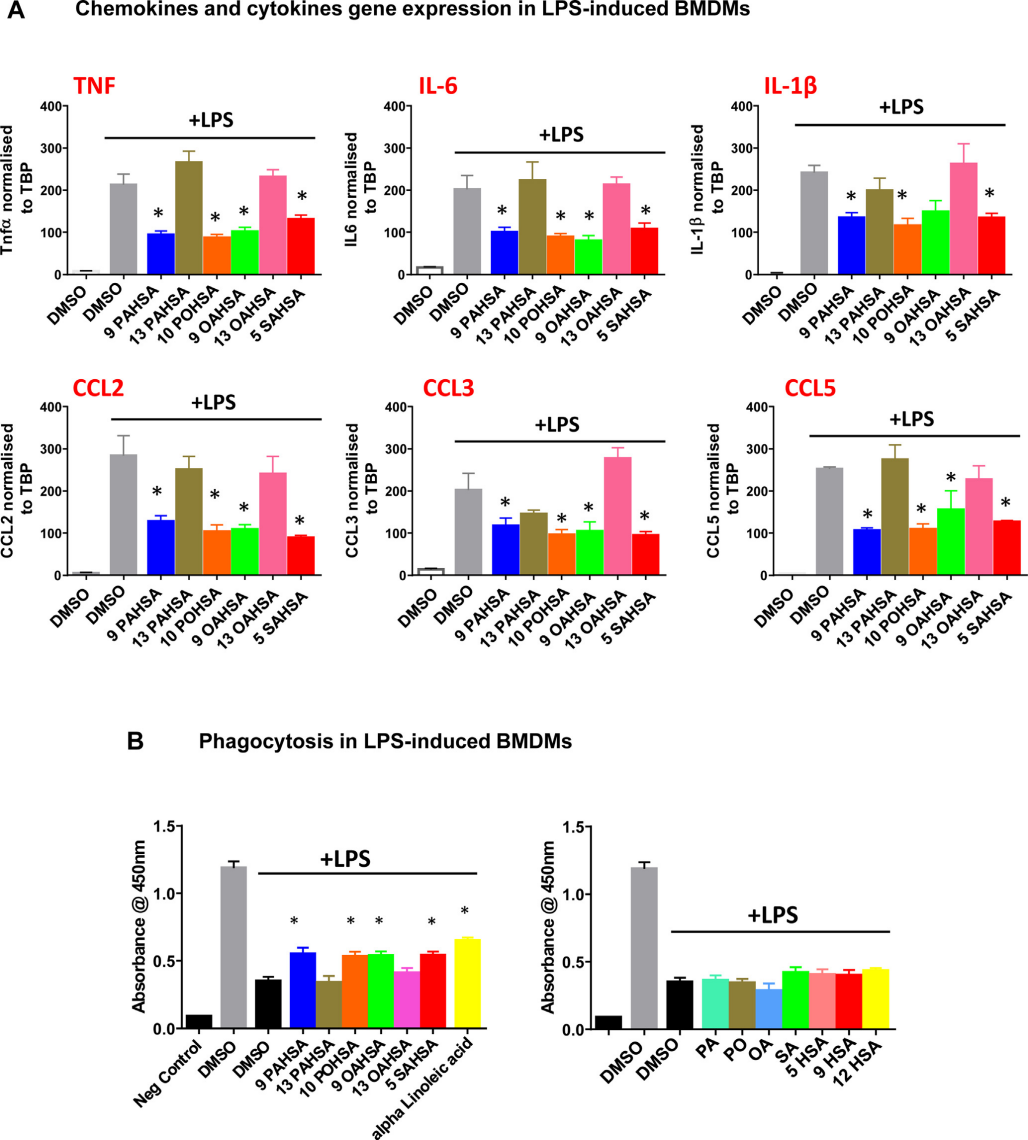
FAHFA isomers inhibit LPS-induced chemokine and cytokine gene expression and stimulate phagocytosis in BMDMs: (A) Various FAHFA isomers (20 μM) inhibit LPS-induced gene expression of different cytokines and chemokines—*Tnf*α, *Il-6*, *Il-1β*, *Ccl2*, *Ccl3*, and *Ccl5*. Data are the means ± SEM; n = 4; ∗*P* < 0.05 versus LPS-induced DMSO by one-way ANOVA. B: Various FAHFA isomers (20 μM) stimulate phagocytosis in BMDMs in the presence of LPS. Data are the means ± SEM. n = 4; ∗*P* < 0.05 versus LPS + DMSO-treated BMDMs by one-way ANOVA. The fatty acids (20 μM), palmitic acid, palmitoleic acid, oleic acid, stearic acid, and hydroxy stearic acids (5-, 9-, and 12-HSA) do not affect phagocytosis in BMDMs. Negative control is macrophages without the addition of zymosan particles. All other bars are different than the negative control. BMDM, bone marrow–derived macrophage; FAHFA, fatty acid ester of hydroxy fatty acid; LPS, lipopolysaccharide.

In BMDMs, LPS treatment increased chemokine and cytokine gene expression. FAHFA isomers, 9-PAHSA, 10-POHSA, and 5-SAHSA, significantly attenuated LPS-induced chemokine *Ccl2*, *Ccl3*, and *Ccl5* and cytokine *Il-1β*, *Tnf-α*, and *Il-6* gene expression (Fig. 6A). 9-OAHSA decreased expression of *Tnfα*, *Il6*, *Ccl2*, *Ccl3*, and *Ccl5* but not *Il-1β*. 13 PAHSA and 13 OAHSA have no anti-inflammatory effects on LPS-induced cytokine and chemokine gene expression. These data are consistent with the effects we observed for individual FAHFAs on secretion of cytokines in BMDMs and BMDCs shown in Fig. 5.

#### Effects of FAHFAs on macrophage phagocytosis

13-DHAHLA enhances the phagocytosis of zymosan particles in macrophages (20). In the present study, we show that other FAHFA isomers 9-PAHSA, 10-POHSA, 9-OAHSA, and 5-SAHSA also improve macrophage phagocytosis in the presence of LPS (Fig. 6B). We used alpha linoleic acid as a positive control because it was previously reported to improve immune cell phagocytosis (33, 34, 35, 36). Effects of FAHFAs on macrophage phagocytosis in the presence of LPS are comparable with those of alpha linoleic acid. 13-PAHSA and 13-OAHSA have no effect on macrophage phagocytosis in the presence of LPS (Fig. 6B). In addition, none of the acyl chain constituents of the FAHFAs we tested have any effect on phagocytosis in macrophages indicating that the intact FAHFA is required for these effects.

### Insulin-stimulated glucose transport

**Fig. 7:**
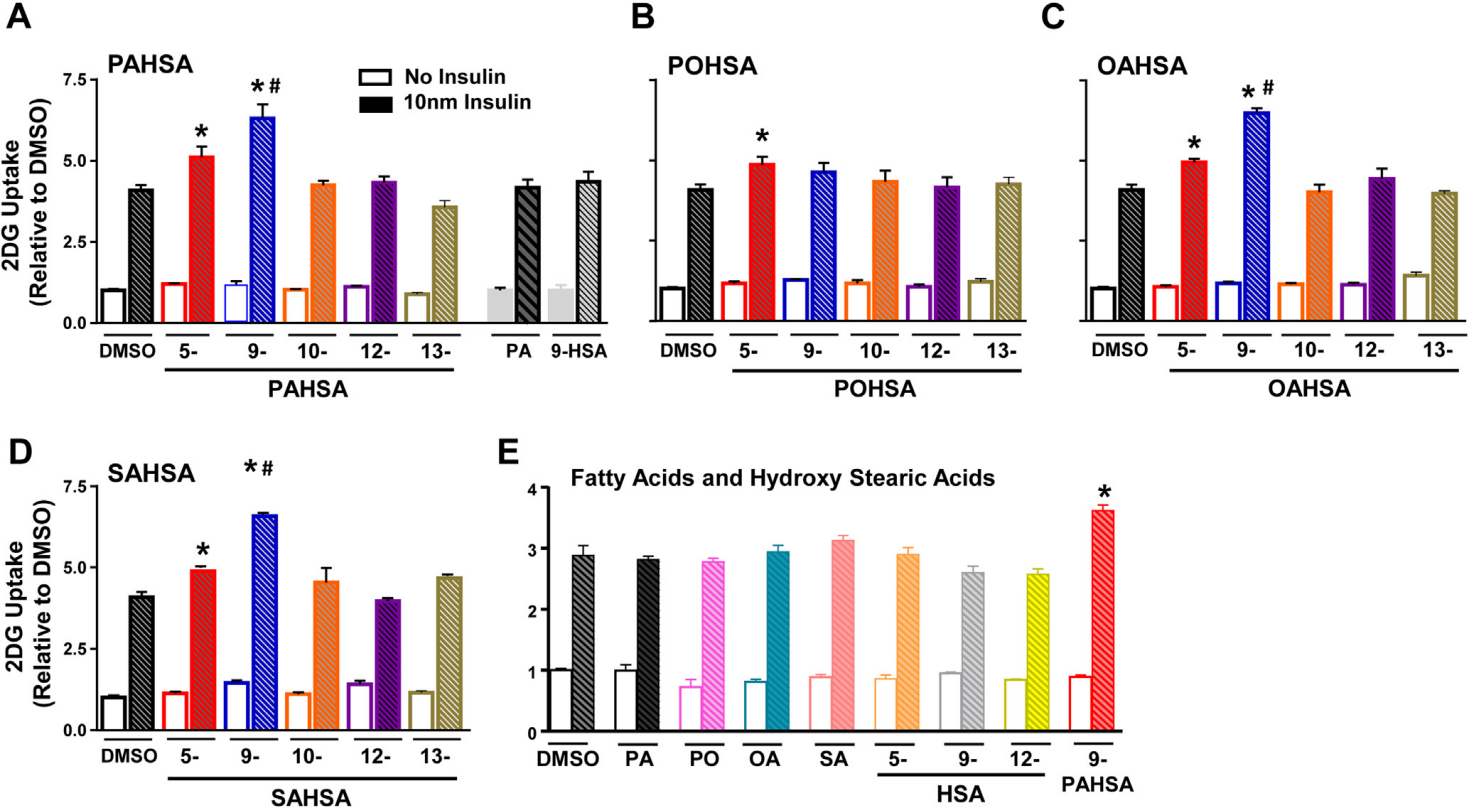
Insulin-stimulated glucose uptake in 3T3 L1 adipocytes. Only 5- and 9-FAHFA isomers potentiate insulin-stimulated glucose transport in 3T3-L1 cells: Differentiated 3T3-L1 adipocytes were treated with FAHFAs (20 μM) or DMSO control for 24 h. Glucose transport in 3T3-L1 adipocytes treated with (A) 5-, 9-, 10-, 12-, and 13-PAHSA, palmitic acid, and 9-hydroxy stearic acid; (B) 5-, 9-, 10-, 12-, and 13-POHSA; (C) 5-, 9-, 10-, 12-, and 13-OAHSA; (D) 5-, 9-, 10-, 12-, and 13-SAHSA. E: Acyl chain constituents of FAHFAs (20 μM), palmitic acid (PA), palmitoleic acid (PO), oleic acid (OA), stearic acid (SA), and hydroxy stearic acids (5-, 9-, and 12-HSA, 20 μM). Data are the means ± SEM. n = 4–6 wells/condition. Each isomer was tested in 2–4 independent studies. ∗*P* < 0.05 versus DMSO; #*P* < 0.05 versus all other conditions. FAHFA, fatty acid esters of hydroxy fatty acid; PAHSA, palmitic acid hydroxy stearic acid; POHSA, palmitoleic acid hydroxy stearic acid; OAHSA, oleic acid hydroxy stearic acid; SAHSA, stearic acid hydroxy stearic acid.

We previously showed that both 5- and 9-PAHSA potentiate insulin-stimulated glucose uptake in 3T3-L1 adipocytes (7). Here, we determined whether other FAHFAs have this effect (Fig. 7). We used a submaximal insulin concentration in 3T3 L1 adipocytes because this is optimal for detecting changes in insulin sensitivity rather than maximal responses. None of the FAHFAs we tested stimulates glucose transport under basal conditions (no insulin) (Fig. 7). At submaximal insulin concentration (10 nM), 5-PAHSA, 9-PAHSA, 5-POHSA, 5-OAHSA, 9-OAHSA, 5-SAHSA, and 9-SAHSA potentiate glucose transport by 20%–60%. 9-PAHSA, 9-OAHSA, and 9-SAHSA potentiate glucose transport more than the corresponding 5-isomers in these families. However, 10-PAHSA, 12-PAHSA, 13-PAHSA, 9-POHSA, 10-POHSA, 12-POHSA, 13-POHSA, 10-OAHSA, 12-OAHSA, 13-OAHSA, 10-SAHSA, 12-SAHSA, and 13-SAHSA do not potentiate insulin-stimulated glucose transport. These data demonstrate that only the 5- and some of the 9-FAHFA isomers potentiate insulin-stimulated glucose transport in 3T3-L1 adipocytes. None of the individual acyl chain constituents of these lipids, palmitic acid, palmitoleic acid, oleic acid, stearic acid, and HSAs (5-, 9-, and 12 HSA) potentiates glucose uptake at either basal or submaximal insulin concentrations (Fig. 7E). The effects of different FAHFAs and their regioisomers are summarized in Table 2.

**Table 2:** Biological activity of different FAHFAs

FAHFA Family	Assay—FAHFA Isomer Compared With DMSO Control	5-FAHFA	9-FAHFA	10-FAHFA	12-FAHFA	13-FAHFA
PAHSA	Insulin-stimulated glucose uptake in adipocytes			↔	↔	↔
	GSIS MIN6 cells				↔	↔
	GSIS human islets				↔	
	Tnfα secretion from BMDCs			↔		
	Il-6 secretion from BMDCs			↔		↔
	Tnfα secretion from BMDMs			↔		↔
POHSA	Insulin-stimulated glucose uptake in adipocytes		↔	↔	↔	↔
	GSIS MIN6 cells	↔	↔			
	GSIS human islets		↔			
	Tnfα secretion from BMDCs				↔	↔
	Il-6 secretion from BMDCs				↔	↔
	Tnfα secretion from BMDMs				↔	↔
OAHSA	Insulin-stimulated glucose uptake in adipocytes			↔	↔	↔
	GSIS MIN6 cells	↔				
	GSIS human islets					
	Tnfα secretion from BMDCs					↔
	Il-6 secretion from BMDCs			↔		↔
	Tnfα secretion from BMDMs			↔	↔	↔
SAHSA	Insulin-stimulated glucose uptake in adipocytes			↔	↔	↔
	GSIS MIN6 cells	↔		↔		
	GSIS human islets					
	Tnfα secretion from BMDCs			↔		↔
	Il-6 secretion from BMDCs			↔		↔
	Tnfα secretion from BMDMs			↔	↔	↔

indicates significant increase compared to DMSO control *P* < 0.05,  indicates significant decrease compared to DMSO *P* < 0.05. ↔ indicates no change.

All assays were repeated 2–5 times, except for GSIS in isolated human islets.

### Metabolic effects of chronic 12-OAHSA treatment in HFD-fed mice

**Fig. 8:**
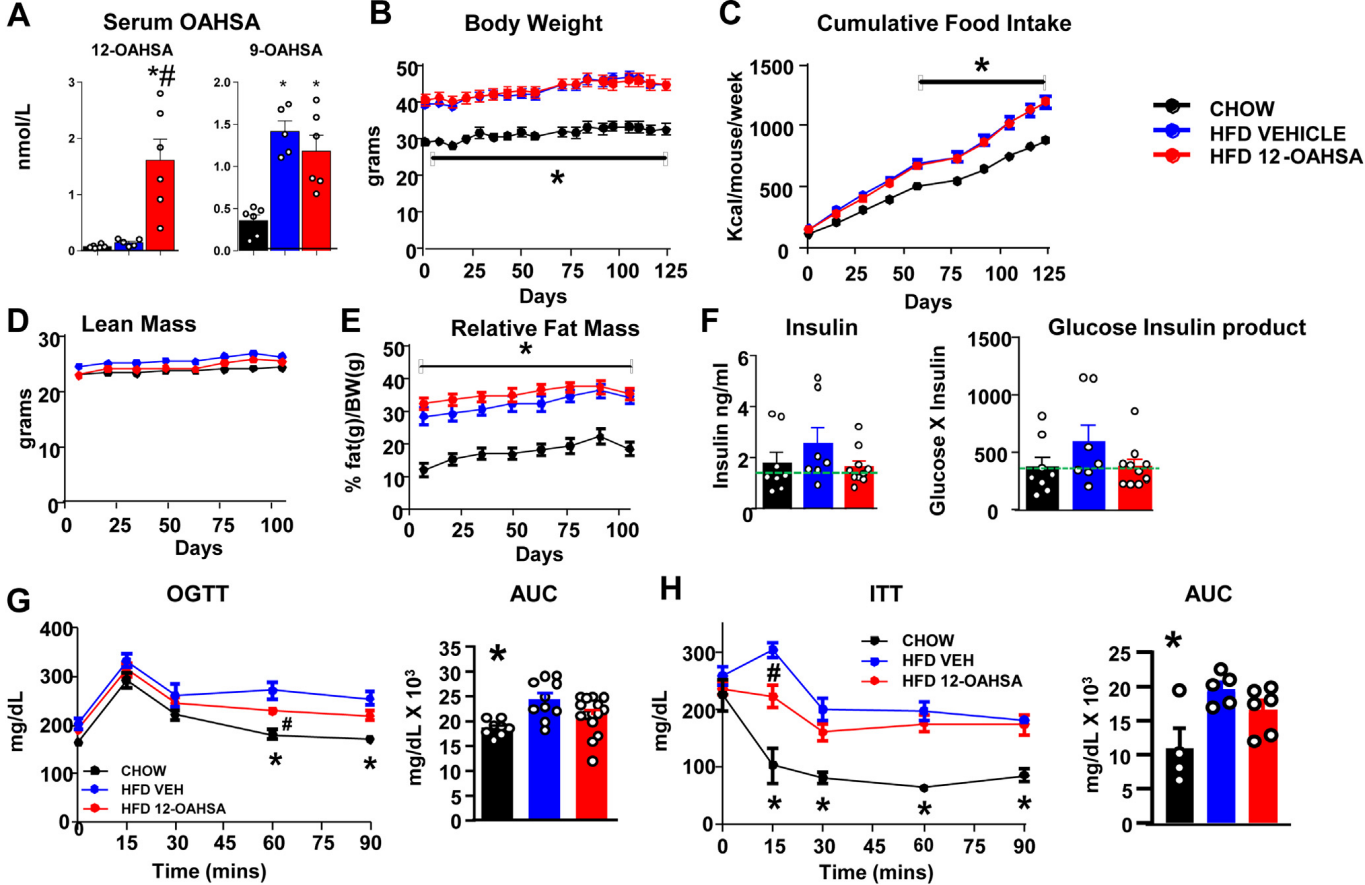
Metabolic effects of chronic 12-OAHSA treatment in HFD-fed mice. (A) Serum 9- and 12-OAHSA levels in chow-fed mice and HFD-fed mice treated with either vehicle or 12-OAHSA via the mini-pump (0.15 μL/h) for 151 days. Data are the means ± SEM. n = 5–6 per group. ∗*P* < 0.05 versus chow, #*P* < 0.05 versus HFD vehicle. (B) Body weight, (C) cumulative food intake, (D) lean mass, (E) relative fat mass in chow-fed and HFD-fed mice treated with vehicle or 12-OAHSA. Data are the means ± SEM. n = 8–10. ∗*P* < 0.05 versus chow. (F) Serum insulin levels and glucose X insulin product in chow- and HFD-fed mice after 151 days of mini-pumps. (G) OGTT in chow- and HFD-fed mice treated with vehicle or 12-OAHSA (57 days of treatment). Data are the means ± SEM. n = 8–15/group; ∗*P* < 0.05 chow versus HFD groups; #*P* < 0.05 HFD 12-OAHSA versus HFD VEH; and (H) ITT in chow-treated, HFD vehicle–treated, and HFD 12-OAHSA-treated mice (122 days of treatment). Data are the means ± SEM. n = 3–6/group. ∗*P* < 0.05 chow versus HFD groups; #*P* < 0.05 HFD 12-OAHSA versus HFD vehicle. ITT, insulin tolerance test; OAHSA, oleic acid hydroxy stearic acid; OGTT, oral glucose tolerance test.

To determine whether a FAHFA family member with relatively low stimulation of GPR40 (Fig. 4A) has similar beneficial effects on glucose homeostasis compared with 5- and 9-PAHSA (7, 13), we treated HFD-fed mice with 12-OAHSA for 22 weeks via an osmotic mini-pump. Serum 12-OAHSA levels increased ∼10-fold with the 12-OAHSA treatment compared with HFD vehicle-treated mice (Fig. 8A), and there was no change in serum 9-OAHSA levels, which we measured as a control. 12-OAHSA treatment in HFD-fed mice had no effect on the body weight (Fig. 8B), cumulative food intake (Fig. 8C), lean body mass (Fig. 8D), or relative fat mass (Fig. 8E). Although there was a range in ad lib–fed serum insulin levels in both vehicle and 12-OAHSA-treated HFD-fed mice, all but one vehicle-treated mice but only half of the 12-OAHSA-treated mice had insulin levels ≥1.32 ng/ml, indicating there could be a slightly higher chance of improved insulin sensitivity with 12-OAHSA treatment. There was no significant difference in ad lib glucose (data not shown) or in the glucose-insulin product (Fig. 8F). Glucose tolerance and insulin sensitivity were slightly improved in 12-OAHSA-treated HFD-fed mice compared with vehicle-treated HFD-fed mice (Fig. 8G, H). Together, these data indicate that chronic 12-OAHSA treatment slightly improves glucose homeostasis in HFD-fed obese mice, but the effects are less than what was reported for 5-PAHSA and 9-PAHSA in the same mouse model (7, 13, 14) or 9-PAHPA and 9-OAHPA in mice on an obesogenic high-fat and high-sugar diet (16, 17).

### Biological activity of 9-PAHSA enantiomers

**Fig. 9:**
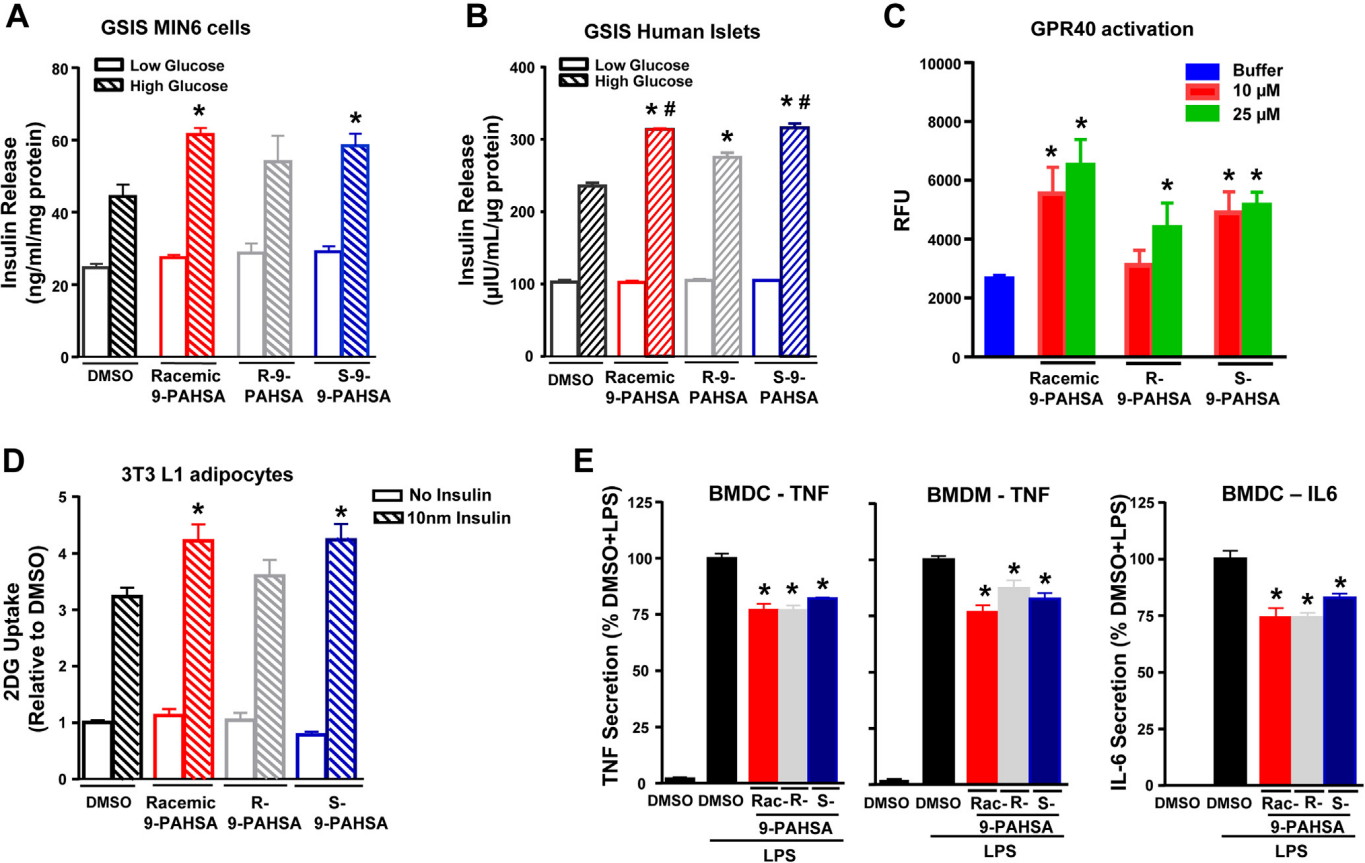
Biological activity of 9-PAHSA enantiomers R- and S-9-PAHSA. A: Insulin secretion from clonal pancreatic MIN6 β-cells: MIN6 cells were incubated with DMSO (control) or racemic, R-9-PAHSA or S-9-PAHSA (20 μM) for 1 h and stimulated with low (2.5 mM) or high (20 mM) glucose for 45 min. n = 8 wells/condition. Each isomer was tested in two independent studies. Data are the means ± SEM. ∗*P* < 0.05 versus high glucose DMSO by one-way ANOVA. B: GSIS from isolated human islets from donor 3. n = 50 islets/well and three wells per each condition. ∗*P* < 0.05 versus high glucose DMSO, #*P* < 0.05 versus high glucose R-9-PAHSA by one-way ANOVA. C: Calcium flux assay in mGPR40 stably transfected cells treated with the control buffer, 9-PAHSA, R-9-PAHSA, and S-9-PAHSA. n = 4/condition. Data are the means ± SEM. ∗*P* < 0.05 versus buffer by one-way ANOVA. D: Basal and insulin-stimulated glucose transport in 3T3L1 adipocytes treated with racemic 9-PAHSA, R-9-PAHSA, S-9-PAHSA (20 μM), or DMSO for 24 h. n = 4–6 wells/condition. ∗*P* < 0.05 versus DMSO. E, Effects of racemic (Rac) and R- and S-9-PAHSA on LPS-induced Tnf or Il-6 secretion from BMDCs and BMDMs. n = 4–6 wells/condition. Each isomer was tested in two independent studies. Data are the means ± SEM. ∗*P* < 0.05 versus LPS-induced DMSO by one-way ANOVA. BMDC, bone marrow–derived dendritic cell; BMDM, bone marrow–derived macrophage; GSIS, glucose-stimulated insulin secretion; LPS, lipopolysaccharide; PAHSA, palmitic acid hydroxy stearic acid.

FAHFAs are chiral at a single carbon and possess either R- or S- forms (enantiomers). Many signaling molecules that work through specific receptors show stereospecificity. Therefore, we tested the biological activity of R- and S-9-PAHSA enantiomers and compared this to the effects of racemic 9-PAHSA on GSIS, glucose transport, and anti-inflammatory effects. In control (DMSO treated) MIN6 pancreatic β cells, a high glucose concentration stimulated insulin secretion by 1.8-fold. Both racemic 9-PAHSA and S-9-PAHSA but not R-9-PAHSA augment GSIS in MIN6 cells compared with DMSO control (Fig. 9A). However, in human islets from normal donors, racemic 9-PAHSA and both R- and S-9-PAHSA potentiate GSIS (Fig. 9B). These studies were carried out in islets from four different healthy donors (donors 3–6; Table 1). Because we have reported that racemic 9-PAHSA effects on GSIS are mediated through GPR40 (13), we tested whether R- and S-9-PAHSA enantiomers activate GPR40. Similar to racemic 9-PAHSA, S-9-PAHSA activates GPR40 at 10 μM and 25 μM concentrations. However, R-9-PAHSA activates GPR40 only at 25 μM (Fig. 9C). The GPR40 activation at lower FAHFA concentrations in this assay compared with the activating concentrations in Fig. 4 or the concentrations we previously reported (13) may reflect a difference in cell density or receptor density on the cells. This is supported by a greater GPR40 activation by the control agonist, linoleic acid, in this experiment compared with the experiment shown in Fig. 4 (data not shown).

Racemic 9-PAHSA potentiates insulin-stimulated glucose uptake at submaximal insulin concentrations in 3T3-L1 adipocytes, as we reported previously (7) (Fig. 9D). Here, we show that S-9-PAHSA also augments insulin-stimulated glucose uptake, but R-9-PAHSA had no effect (Fig. 9D). Figure 9E shows that racemic 9-PAHSA, R-9-PAHSA, and S-9-PAHSA all exert anti-inflammatory effects as measured by LPS-induced Tnf-α secretion from BMDCs and BMDMs and Il6 secretion from BMDCs. Thus, some biologic effects of PAHSAs show stereospecificity, but others do not. The effects of the 9-PAHSA stereoisomers are shown in Table 3.

**Table 3:** Biological activity of racemic 9-PAHSA and 9-PAHSA enantiomers

Assay		Racemic 9-PAHSA Versus DMSO (n)	R-9-PAHSA Versus DMSO (n)	S-9-PAHSA Versus DMSO (n)
Insulin-stimulated glucose uptake in adipocytes		(4)	↔ (4)	(4)
GSIS MIN6 cells		(2)	↔ (2)	(2)
GSIS human islets		(4)	(4)	(4)
GPR40 activation assay	10 μM PAHSA	(1)	↔ (1)	(1)
Calcium flux	25 μM PAHSA	(1)	(1)	(1)
Tnfα secretion from BMDCs		(2)	(2)	(2)
Il-6 secretion from BMDCs		(2)	(2)	(2)
Tnfα secretion from BMDMs		(2)	(2)	(2)

indicates significant increase compared to DMSO control *P* < 0.05,  indicates significant decrease compared to DMSO *P* < 0.05. ↔ indicates no change.

The number of assays performed is given in parentheses.

## Discussion

Recently, an increasing number of FAHFA families and hundreds of regioisomers have been identified in humans, animals, and plants (7, 8, 9, 11). However, the biological functions of only a few FAHFA family members have been reported (7, 13, 14, 16, 20, 21, 22, 23, 27, 37, 38, 39, 40). We sought to determine whether different isomers within the same FAHFA family and whether FAHFAs from different families have similar biological effects. Here, we investigated the biological activities of 5-, 9-, 10-, 12-, and 13-isomers of the FAHFA family members PAHSAs, POHSAs, OAHSAs, and SAHSAs on GSIS from human islets and clonal pancreatic β cells, insulin-stimulated glucose uptake in adipocytes, and anti-inflammatory effects in BMDCs and BMDMs.

We found that several FAHFAs that have not been tested before potentiate GSIS in MIN6 cells and human islets similar to 5- and 9-PAHSA. In the OAHSA family, the extent of potentiation of GSIS appears to depend on the position of the ester bond between the oleic acid and HSA moieties. OAHSA isomers with branching distal to the carboxylate head group appear to be most efficient in potentiating insulin secretion although this is not apparent for other FAHFA families. In the POHSA family, 10-, 12-, and 13-isomers potentiate GSIS, suggesting in FAHFAs containing an unsaturated acyl chain, GSIS is stimulated by isomers with the ester bond at a higher carbon position.

Because we previously showed that the augmentation of GSIS in Min6 cells and human islets is mediated by GPR40 and inhibition of GPR40 reduces the beneficial effects of 9-PAHSA on GSIS, glucose tolerance, and insulin sensitivity in vivo (13), we tested GPR40 activation with other FAHFA members. Most but not all FAHFAs tested activate GPR40, and this is not due to activation by the individual acyl chain constituents of FAHFAs. In the in vitro GPR40 activation assay, we saw effects at 50 and/or 100 μM (Fig. 4), while most of the FAHFA biological activities are present at a concentration of 20 μM. The GPR40 assay is performed in stably transfected HEK293 cells that were engineered to have a low receptor copy number to distinguish partial from total GPR40 agonists. The discrepancy between the FAHFA concentrations that activate GPR40 in this assay compared with the concentrations that activate insulin secretion, glucose transport, and anti-inflammatory effects in the relevant cells might be due to a difference in the GPR40 receptor density on the HEK cells compared with the target cells for the biological assays. It could also be due to the absence of other proteins and molecules or cofactors that modulate GPR40 activation or FAHFA biologic effects in target cells. We found activation of GPR40 at lower PAHSA concentrations in the assay performed with racemic 9-PAHSA and 9-PAHSA stereoisomers (Fig. 9C). This could be explained by a difference in cell density or receptor density between the studies, which was indicated by a greater response of the positive control agonist, linoleic acid (data not shown), in the study in Fig. 9C. However, the data are useful for comparing FAHFA isomers to each other within each of the studies.

Chronic, low-grade inflammation in adipose tissue plays an important role in obesity-related insulin resistance (41, 42). Pathogen-derived molecules, such as LPS, and saturated fatty acids, such as palmitic acid, promote BMDC maturation by activating toll-like receptors. This further induces expression of proinflammatory cytokines including *Il-1β*, *Il-6*, and *Tnf-α*, and chemokines including *Ccl2*, *Ccl3*, and *Ccl5* required for antigen presentation, T cell activation, and migration. Here, we demonstrate that multiple isomers from all four FAHFA families we tested exert anti-inflammatory effects similar to the published effects of 5- and 9-PAHSA. Most of these FAHFA isomers have anti-inflammatory effects in both BMDMs and BMDCs. However, a few isomers exert anti-inflammatory effects only in BMDCs but not in BMDMs, suggesting that the anti-inflammatory effects of some FAHFAs are immune cell specific. Furthermore, all the 5- and 9-FAHFA isomers tested attenuated LPS-induced Tnf-α secretion in BMDCs and BMDMs, and Il-6 secretion in BMDCs suggesting that FAHFAs with lower branching from the carboxylate head group more consistently have anti-inflammatory properties compared with those with higher branching from the carboxylate head group. In addition, most FAHFA isomers attenuate LPS-induced chemokine and cytokine secretion (Fig. 5C) and gene expression (Fig. 6A) in macrophages, suggesting that FAHFAs exert transcription effects on inflammation that would attenuate the innate immune response. A recent article showed that the omega-3 fatty acid–containing FAHFA, 13-DHAHLA, enhances the phagocytosis of zymosan particles by macrophages (20). We found that most FAHFAs we studied also enhance phagocytotic activity in macrophages (Fig. 6B). None of the individual acyl chain constituents of the FAHFAs tested have any anti-inflammatory effects in our assay system.

5- and 9-PAHSA augment insulin-stimulated glucose uptake (7, 38, 43, 44), Glut4 translocation to the plasma membrane (7, 43), and specifically Glut4 exocytosis (43) in 3T3-L1 adipocytes and freshly isolated mouse adipocytes. However, nothing has been reported on the effects of other FAHFA family members on potentiating insulin-stimulated glucose uptake. Here, we demonstrate that several other FAHFA family members potentiate insulin-stimulated glucose uptake. Notably, all 5- and 9-isomers of the families we tested, except 9-POHSA, increase glucose uptake in 3T3-L1 adipocytes. Furthermore, the 9-isomers of PAHSA, OAHSA, and SAHSA, but not 9-OAHSA, increase glucose uptake to a greater extent than the corresponding 5-isomers. In addition, FAHFAs containing either saturated or unsaturated fatty acids augment glucose transport, indicating that the degree of unsaturation does not affect the ability of FAHFAs to stimulate glucose transport.

Before this study, we had investigated beneficial effects of only 5- and 9-PAHSAs on glucose homeostasis. In this study, we show that 12-OAHSA slightly improves glucose tolerance and insulin sensitivity in HFD-fed mice, with no change in the body weight, fat mass, or food intake. The effect is not as great as we reported with 5- and 9-PAHSA (7, 13, 14) or as others reported with 9-PAHPA and 9-OAHPA (16, 17) in diet-induced obese mice. These data suggest that not all the FAHFA family members have similar beneficial effects on improving glucose homeostasis.

Biological systems usually discriminate among different enantiomers of chiral compounds (45). There is limited published data on the R and S enantiomers of FAHFAs (25, 26, 27). Here, we show that both racemic 9-PAHSA and S-9-PAHSA but not R-9-PAHSA augment glucose-simulated insulin secretion in MIN6 cells compared with the DMSO control. However, in human islets, racemic 9-PAHSA and both its enantiomers potentiated GSIS compared with the DMSO control. The potentiation of S-9-PAHSA is similar to racemic 9-PAHSA, whereas the effect of R-9-PAHSA is lower. In addition, racemic and S-9-PAHSA augment insulin-stimulated glucose transport but R-9 PAHSA does not. However, both R-9-PAHSA and S-9-PAHSA show similar anti-inflammatory effects in LPS-activated BMDCs and BMDMs. Paluchova *et al.* showed similar anti-inflammatory effects of 13 R- and 13 S-DHAHLA to inhibit antigen- and PGE2-induced chemotaxis and degranulation of mast cells. Thus, there is stereospecificity for some effects of 9-PAHSA but not for all effects.

The different results with different biological assays may reflect the fact that these biological activities are mediated by distinct G protein–coupled receptors (GPCRs) (7, 13, 14). At least three different GPCRs have been identified that mediate distinct biological effects of 5- and 9-PAHSA: G protein-coupled receptor 120 for the glucose transport effects (7), GPR40 for the insulin secretion (13), and a Gαi receptor for the effects on hepatic glucose production (14). Undoubtedly, other receptors are involved in other effects of FAHFAs. We sought to determine whether differences in the activation of GPR40 may play an important role in mediating the stereospecific effects of 9-PAHSA enantiomers on augmentation of GSIS. This does not seem to be the case because R- and S-9-PAHSA activate GPR40 similarly at a concentration of 25 μM, which is similar to the concentration used in the assays of biologic activity. S-9 PAHSA and racemic 9-PAHSA both stimulated GPR40 at 10 μM, while R-9PAHSA did not, but the biological assays were not performed at this concentration. More studies would be required to determine whether this reflects a difference in affinity of the stereoisomers for GPR40.

We previously observed stereospecificity in the hydrolysis of FAHFAs by CEL (25), consistent with the fact that the FAHFA stereocenter is being hydrolyzed by a chiral enzyme. By contrast, the absence of stereospecificity for the anti-inflammatory effects of FAHFAs indicates that the stereocenter is not an important component of target protein binding or that the target protein(s) can engage both stereoisomers. Activity with both stereoisomers is observed with other lipids as well. For example, the hydroxy fatty acids 15R-hydroxyeicosatetraenoic acid and 15S-hydroxyeicosatetraenoic acid are equipotent activators of PPARδ (46), and 9R-hydroxyoctadecadienoic acid and 9S-hydroxyoctadecadienoic acid are agonists with similar potency with PPARγ (47). The ability of stereoisomers of hydroxy fatty acids and FAHFAs to show similar activity is likely due to the flexibility (“floppiness”) of lipids that allows them to contort into the necessary shape to activate their receptors of different isomers. In addition, in FAHFAs, the branching occurring after the ester bond presents two lipophilic chains that can create pseudo-achirality when considering potential intermolecular binding.

In conclusion, FAHFA variants and isomers within FAHFA families display differences in biological activities, suggesting that different FAHFAs have different physiological functions. In this study, we found that only a few FAHFAs have all the biological activities we tested. More FAHFAs have effects on GSIS and cytokine secretion compared with insulin-stimulated glucose uptake. This suggests that FAHFAs display cell-specific biological activity. Because multiple GPCRs mediate the effects of FAHFAs, the difference in the effects of specific FAHFAs on these biological activities may reflect the involvement of different GPCRs.

Within a FAHFA family, isomers with lower branching from the carboxylate head group are more likely to be anti-inflammatory. In contrast, for FAHFAs containing unsaturated acyl chains, isomers with higher branching from the carboxylate group are more likely to potentiate GSIS in clonal β-cells. Neither the saturated nor the unsaturated acyl chains of FAHFAs alone showed biological activity in the assays we used. Therefore, the FAHFAs need to be intact to exert these effects. In addition, some biological activities of 9-PAHSA are stereospecific (e.g., insulin-stimulated glucose transport), whereas others (e.g., anti-inflammatory effects and GSIS in human islets) are not. This study provides insight into the specificity or generality of different FAHFA families and their isomers on biological actions that have been shown to be important in disease states. These studies could lead to the development of individual FAHFAs or combinations to treat metabolic and immune-mediated diseases.

## Data availability

All study data are included in the article.

## Conflict of interest

B. B. K., A. S., and I. S. are inventors of the following patents related to the fatty acid hydroxy fatty acid: “Lipids that Increase Insulin Sensitivity and Methods of Using the same” (patent no. 20180194714) and “Fatty Acid Esters of Hydroxy Fatty Acids (FAHFAs) for Use in the Treatment of Type 1 Diabetes” (patent no. 20190151276); B. B. K., A. S., I. S., and J. L. are inventors of the following patent related to the fatty acid hydroxy fatty acid “Methods of Preventing and Treating Inflammatory Bowel Disease with Branched Fatty Acid Esters of Hydroxy Fatty Acids (FAHFAs)” (patent application no. WO2017070515A2). All other authors have no known competing financial interests or personal relationships that could have appeared to influence the work reported in this article.
